# Inflammatory cell infiltration in left atrial appendageal tissues of patients with atrial fibrillation and sinus rhythm

**DOI:** 10.1038/s41598-020-58797-8

**Published:** 2020-02-03

**Authors:** Christopher Hohmann, Roman Pfister, Martin Mollenhauer, Christoph Adler, Jolanta Kozlowski, Andreas Wodarz, Uta Drebber, Jens Wippermann, Guido Michels

**Affiliations:** 1 Department III of Internal Medicine, Heart Center, University of Cologne, Faculty of Medicine and University Hospital of Cologne, Kerpener Str. 62, 50937 Cologne, Germany; 2 Center for Molecular Medicine Cologne, University of Cologne, Kerpener Str. 62, 50937 Cologne, Germany; 3 Molecular Cell Biology, Institute I for Anatomy, University of Cologne Medical School, Joseph-Stelzmann Str. 9, 50931 Cologne, Germany; 4 Cluster of Excellence – Cellular stress response in aging-associated diseases (CECAD), Joseph-Stelzmann Str. 26, 50931 Cologne, Germany; 5 Institute for Pathology, University of Cologne, Faculty of Medicine and University Hospital Cologne, Kerpener Str. 62, 50937 Cologne, Germany; 6 Department of Cardiac Surgery, University Hospital of Magdeburg, Leipziger Str. 44, 39120 Magdeburg, Germany

**Keywords:** Cardiology, Diseases, Pathogenesis

## Abstract

Atrial fibrillation (AF) is the most common sustained cardiac arrhythmia in clinical practice and is known to be associated with significant morbidity and mortality. Previous studies suggested a link between inflammation and AF by findings of increased inflammatory markers in AF patients. However, it has not been finally clarified whether inflammation is a systemic or a local phenomenon reflecting an active inflammatory process in the heart. To address this subject, human left atrial appendage tissues were obtained from 10 patients who underwent cardiac surgery and subjected to immunohistochemical analysis. The number of inflammatory CD3-positive T cells significantly increased from patients with sinus rhythm to paroxysmal AF and persistent AF, respectively. Interestingly, in patients with persistent AF, these cells were frequently arranged in small clusters. Subsequently, the number of inflammatory CD3-positive T cells decreased and was significantly lower in patients with permanent AF than in patients with persistent AF. Inflammatory CD20-positive B cells could only be detected very occasionally in all AF subgroups and were not locatable in patients with SR. Hence, our data emphasize the potential prominent role of the cellular component of the immune system in the development and perpetuation of AF.

## Introduction

Atrial fibrillation (AF) is the most common sustained supraventricular arrhythmia worldwide and is associated with considerable morbidity and mortality. Approximately every fifth ischemic stroke is caused by this arrhythmia and the all-cause mortality of patients suffering from AF is about 2-fold increased in women and 1.5-fold in men^1^. In 2010, about 33.5 million adults were estimated to have AF worldwide, with a prevalence predicted to further increase until 2060^2^. The main conditions predisposing to AF are aging, arterial hypertension, coronary artery disease, congestive heart failure and valvular heart disease. Additionally, extracardiac factors such as diabetes mellitus, thyroid disease, obesity, obstructive sleep apnea and heavy alcohol consumption are known factors that contribute to the development of this arrhythmia^3,4^. However, approximately 10% of all AF cases are suspected to be idiopathic without confirmation of any aforementioned conspicuity^5^.

Clinical AF can be divided into mainly three different forms: paroxysmal, which typically ceases within 48 hours or at least after 7 days; persistent, which lasts longer than 7 days and/or requires antiarrhythmic drug treatment or electrical cardioversion to terminate; and permanent. In short, focal sources particularly around the pulmonary veins generate paroxysmal AF forms^6^. The induction of the arrhythmia is precipitated by several modulating and trigger factors such as vegetative stimulations, extrasystoles, or acute atrial stretch. The emergence of functional reentry substrates causes persistent AF that can be terminated in order to restore normal sinus rhythm. The maintenance of AF and the permanent form is undertaken by atrial remodeling, which is a multifactorial process containing structural, electrophysiological and molecular pattern^7^. Main factors include pulmonary venous dilatation, atrial myocardial stretch, interstitial fibrosis, myocytic hypertrophy and degeneration, changes in sarcoplasmic reticulum calcium homeostasis, oxidative stress, altered expression of ion channels and microvascular dysfunction^8^.

Despite the clinical relevance of this arrhythmia, the exact underlying pathogenesis of AF remains only partly understood to the present day. In the past, several findings have emphasized the influence of inflammation in the development of the disease^9^. For instance, epidemiologic and clinical studies have shown an association between several inflammatory markers- such as C-reactive protein (CRP), tumor necrosis factor alpha (TNFα) or interleukin 6 (IL-6)- and both the presence of AF and the risk of developing AF in the future^10–12^. More importantly, anti-inflammatory therapies with statins, steroids or vitamin C led to a substantial decrease of AF episodes^13–16^. However, there have only been few previous histological surveys analyzing the association between inflammation and AF with regard to elevated inflammatory cell counts in human tissue samples^17–19^. It is important to note in this respect that these studies partially only relied on material from bioptic samples or right atrial appendage tissue and that analysis of distinct immune cell populations differed between the different assays. Furthermore, data regarding immunhistochemical analysis of various stages of AF and sinus rhythm (SR) are generally lacking. Therefore, in our study, we aimed to investigate the association of inflammatory B- and T-cell infiltration in left atrial appendages tissues of patients with paroxysmal, persistent and permanent AF in comparison to SR. Additionally, we tended to correlate the presence of atrial immune cells populations with markers of systemic inflammation.

## Materials and Methods

### Patients

In our monocentric, prospective observational study, left atrial appendages were obtained from patients undergoing coronary artery bypass surgery, mitral valve repair or replacement, tricuspid valve repair, aortic valve replacement, surgical bipolar AF ablation or any combination thereof between 2013 and 2018. The study included 2 patients with SR, 2 patients with paroxysmal AF, 3 patients with persistent AF and 3 patients with permanent AF, respectively. In patients with SR, left atrial appendages were excised in case of considerably dilated left atria and the presence of ≥3 risk factors for the development of atrial fibrillation. Clinical history and laboratory findings, especially serum markers of inflammation (leukocytes, C-reactive protein, IL-6) were collected before surgery. Patients who presented with sepsis, permanent cardiostimulation, previous myocardial infarction, febrile disorder, infectious or inflammatory disease, autoimmune disease, or use of immunosuppressive drugs were excluded. The heart rhythm was ascertained from electrocardiograms just before the operation. This investigation conformed to the principles presented in the Declaration of Helsinki and was approved by the Ethics Committee of the medical faculty at the University Hospital of Cologne (vote 12–226). Informed consent was obtained from all study subjects.

### Tissue sampling and histological analysis

Whole left atrial appendages were obtained during open-heart surgery and incisions were continuously sutured. Tissues were immediately frozen in liquid nitrogen after resection. Frozen cryostat sections (8-µm thick) were cut, air-dried, fixed in acetone and then evaluated with standard protocols for staining with hematoxylin and eosin.

### Immunohistochemical analysis

**Figure 1:**
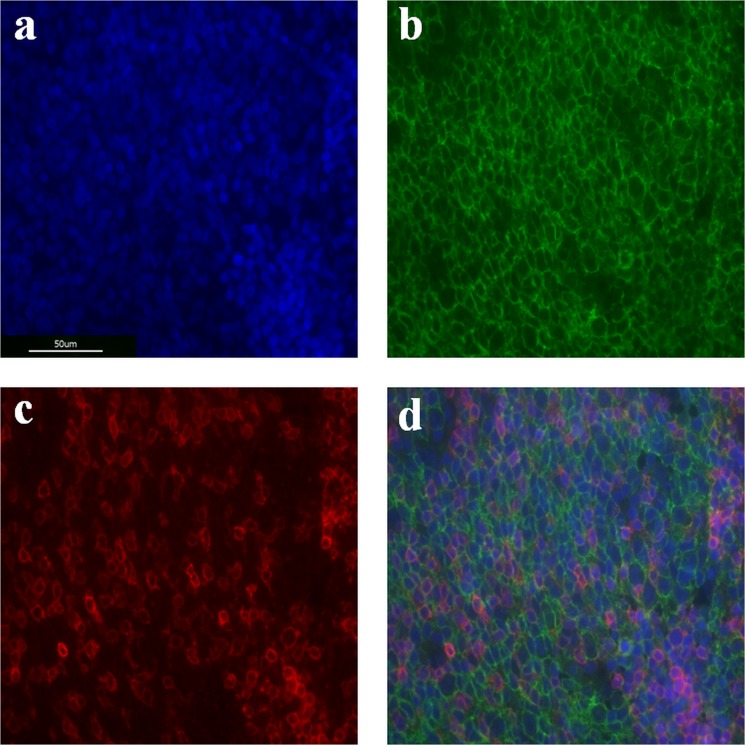
Immunofluorescent stainings of human tonsil samples for positive control. The DNA binding dye (DAPI, blue) enables visualization of the cellular nucleus, and thereby the cell distribution (**a**). Localization of CD20-positive B cells (**b**) and CD3-positive T cells (**c**) with overlapping expressions (**d**) are presented.

Paraffin-embedded, 2 µm-thick tissue sections were deparaffinized with xylene and rehydrated through decreasing concentrations of alcohol. For antigen retrieval, tissue sections were heated at 95 °C in 0.01 mM citric buffer (Citrate Buffer pH 6.0 ZUC028–500, Zytomed, Berlin, Germany) for 15 minutes, then quenched for 40 minutes and rinsed in 0.05 mM TBS buffer. Nonspecific background was eliminated using 5% BSA solution in TBS for 60 minutes (Albumin Fraction V, Roth, Karlsruhe, Germany). Primary mouse monoclonal antihuman CD 20 antibody (Abcam, Cambridge, UK) and primary rabbit monoclonal antihuman CD 3 antibody (Abcam, Cambridge, UK) were used at a dilution of 1 in 50 and 1 in 100, respectively, and applied to sections overnight at 4 °C in 0.8% BSA solution in TBS. The sections were then washed thrice with TBS buffer and incubated with secondary antibodies (Goat anti-mouse, 1:500, Invitrogen, Carlsbad, California; Goat anti-rabbit, 1:500, Invitrogen, Carlsbad, California; and Hoechst, 1:100, Biotium, Fremont, California) for 60 minutes at room temperature. Tissue sections were then washed thrice with TBS buffer and finally cover sections were mounted with Aqua-Poly-Mount (Polyscience, Niles, Illinois). Human tonsil tissue was provided by the Institute for Pathology of the University Hospital of Cologne and served as a positive control (Fig. 1).

### Histomorphometry

To quantify CD 20+ cells and CD3+ cells we used the program Image J 1.52a (National Institutes of Health, USA). Images for quantification were collected by systematic uniform random sampling of tissue sections using the 60x objective with immersion oil of a Keyence BZ-X800 microscope (Keyence, Neu-Isenburg, Germany). From each sample of left atrial appendage tissue, 60 images from a total of two sections were recorded. Individual recordings were separated from each other by 1 image frame. Only immune cells within the myocardium were counted manually and labeled in the image analysis program to make sure that no cell is counted twice. The frequency of cells was expressed as the number of cells per square millimeter. Those who performed image analysis were blinded to patient characteristics.

### Statistical analysis

Data were expressed as means ± SD or percentages. Variables between the AF groups and the sinus group were compared using one-way analysis of variance (ANOVA). Additionally, when appropriate in case of significant differences, the Tukey post-hoc test was applied. All statistical analyses were performed using SPSS software, version 24.0 (SPSS Inc., Chicago, USA). Statistical significance was set at a p-value of <0.05.

## Results

### Baseline characteristics of patients studied

Baseline clinical characteristics of the study patients are presented in Table 1. The 4 groups (SR, paroxysmal AF, persistent AF, and permanent AF) did not differ in age, gender, heart failure status, or with regard to structural heart disease. However, there was a tendency towards a larger area of the left atrium in patients with AF. With respect to laboratory values, we could only observe significant differences in serum triglycerides between patients with paroxysmal and persistent AF (p = 0.032) with on average higher maximum values measured in the subgroup of paroxysmal AF. Interestingly, inflammatory markers (C-reactive protein, leukocytes, interleukin-6) did not significantly differ neither between patients with SR or AF nor within the different subgroups of AF. However, there was a tendency towards higher levels of CRP and IL-6 in patients with persistent and permanent AF, respectively.

**Table 1:** Baseline clinical characteristics of patients studied.

	Sinus rhythm (n = 2)	Paroxysmal AF (n = 2)	Persistent AF (n = 3)	Permanent AF (n = 3)	p Value
**Patient demographics**					
Age (yrs)	73.5 ± 7.8	72.5 ± 13.4	68.0 ± 3.0	69.0 ± 6.2	0.822
Male (%)	1 (50)	1 (50)	2 (66.6)	2 (66.6)	
**Cardiac measurements**					
Left atrial area (cm²)	19.0 ± 7.1	25.8 ± 8.7	23.0 ± 8.7	29.4 ± 20.2	0.818
Right atrial area (cm²)	18.9 ± 4.3	17.8 ± 0.7	21.7 ± 5.1	23.0 ± 11.8	0.864
Left ventricular ejection fraction (%)	62.0 ± 5.7	57.5 ± 17.7	53.3 ± 17.5	56.0 ± 1.0	0.897
Heart rate at baseline (bpm)	68.0 ± 8.5	69.5 ± 7.8	70.0 ± 8.5	71.3 ± 4.7	0.676
**Laboratory findings**					
Sodium (mmol/l)	138.5 ± 3.5	133.0 ± 0.0	139.7 ± 5.1	141.3 ± 1.1	0.146
Potassium (mmol/l)	3.8 ± 0.2	4.9 ± 2.4	4.2 ± 0.5	4.3 ± 0.3	0.794
Magnesium (mmol/l)	0.85 ± 0.06	0.94 ± 0.03	0.84 ± 0.02	0.84 ± 0.07	0.262
Creatinine (mg/dl)	1.27 ± 0.49	1.80 ± 0.22	1.06 ± 0.10	1.52 ± 0.68	0.379
Trigylceride (mg/dl)	133.0 ± 1.4	243.5 ± 95.5	77.0 ± 15.9	186.7 ± 28.3	0.024
Total cholesterol (mg/dl)	164.5 ± 29.0	222.0 ± 18.4	155.3 ± 32.3	187.3 ± 4.9	0.085
LDL cholesterol (mg/dl)	89.0 ± 24.0	104.0 ± 19.8	95.7 ± 27.3	115.0 ± 8.5	0.563
HDL cholesterol (mg/dl)	55.0 ± 4.2	80.5 ± 23.3	47.7 ± 13.1	43.7 ± 9.0	0.091
TSH (ng/l)	1.38 ± 0.49	3.21 ± 0.70	0.88 ± 0.94	3.52 ± 2.70	0.284
T3 (ng/l)	2.80 ± 0.42	3.50 ± 0.71	3.30 ± 0.56	3.00 ± 0.36	0.537
T4 (ng/l)	12.80 ± 2.97	14.55 ± 1.06	13.23 ± 1.61	13.97 ± 1.02	0.726
Leukocytes (×10^9^/l)	8.30 ± 0.72	5.76 ± 0.13	6.30 ± 1.09	7.77 ± 2.78	0.438
CRP (mg/l)	0.69 ± 0.44	1.70 ± 0.99	5.87 ± 8.43	4.53 ± 3.29	0.689
Interleukin-6 (ng/l)	5.00 ± 2.83	4.50 ± 0.71	6.30 ± 1.09	7.77 ± 2.78	0.383
**Concomitant medications**					
Acetylsalicylic acid (%)	2 (100)	1 (50)	1 (33.3)	3(100)	
ACE inhibitors (%)	2 (100)	1 (50)	1 (33.3)	1 (33.3)	
β- Blocker (%)	2 (100)	2 (100)	3 (100)	3 (100)	
Statins (%)	2 (100)	2 (100)	3 (100)	3 (100)	
Oral anticoagulation (%)	1 (50)	0 (0)	2 (66.6)	2 (66.6)	
NSAIDs (%)	0 (0)	0 (0)	0 (0)	0 (0)	
Steroids (%)	0 (0)	0 (0)	0 (0)	0 (0)	
Diuretics (%)	1 (50)	2 (100)	3 (100)	1 (33.3)	
**Preoperative heart disease**					
Valvular heart disease (%)	1 (50)	2 (100)	0 (0)	2 (66.6)	
Ischemic heart disease (%)	1 (50)	0 (0)	3 (100)	1 (33.3)	
**Surgical treatment**					
Patient I	Aortic valve replacement, mitral valve replacement, coronary artery bypass	Mitral valve repair, surgical AF ablation	Coronary artery bypass, surgical AF ablation	Mitral valve replacement, surgical AF ablation	
Patient II	Mitral valve repair	Mitral valve replacement, coronary artery bypass	Coronary artery bypass, surgical AF ablation	Coronary artery bypass, surgical AF ablation	
Patient III			Coronary artery bypass, surgical AF ablation	Mitral valve repair, trikuspid valve repair, surgical AF ablation	

### Increased number of inflammatory T lymphocytes in the fibrillating atria

**Figure 2:**
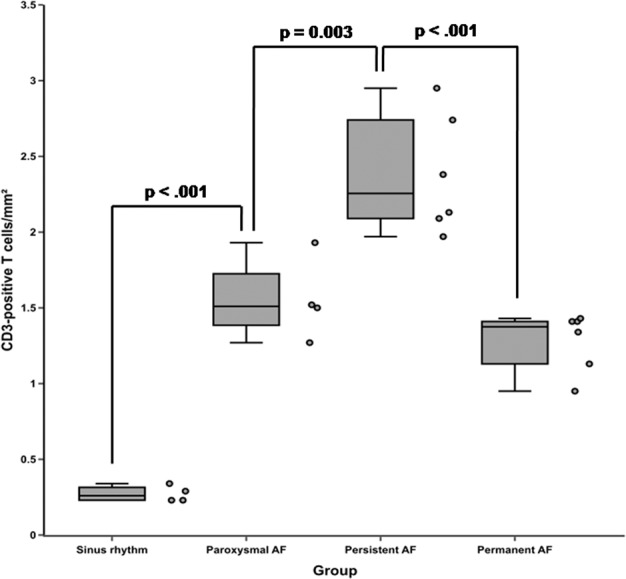
Frequency of CD3-positive T cells in left atrial appendage tissues of patients with sinus rhythm (SR) or paroxysmal, persistent and permanent atrial fibrillation (AF). Box-plots presenting median and interquartile range of CD3-positive T cells per square mm are given. Adjacent dot plots demonstrate data distribution in each subgroup. The number of inflammatory CD3-positive T cells was significantly higher in patients with paroxysmal AF than in the SR group (p < 0.001) and significantly higher in patients with persistent AF than in the subgroup of paroxysmal AF (p = 0.003). Subsequently, the number of inflammatory CD3-positive T cells decreased and was significantly lower in patients with permanent AF than in patients with persistent AF (p < 0.001). Variables between the AF groups and the sinus group were compared using one-way analysis of variance (ANOVA). From each patient and sample of left atrial appendage tissue, a total of two sections with respectively 30 images were recorded and analyzed.

**Figure 3:**
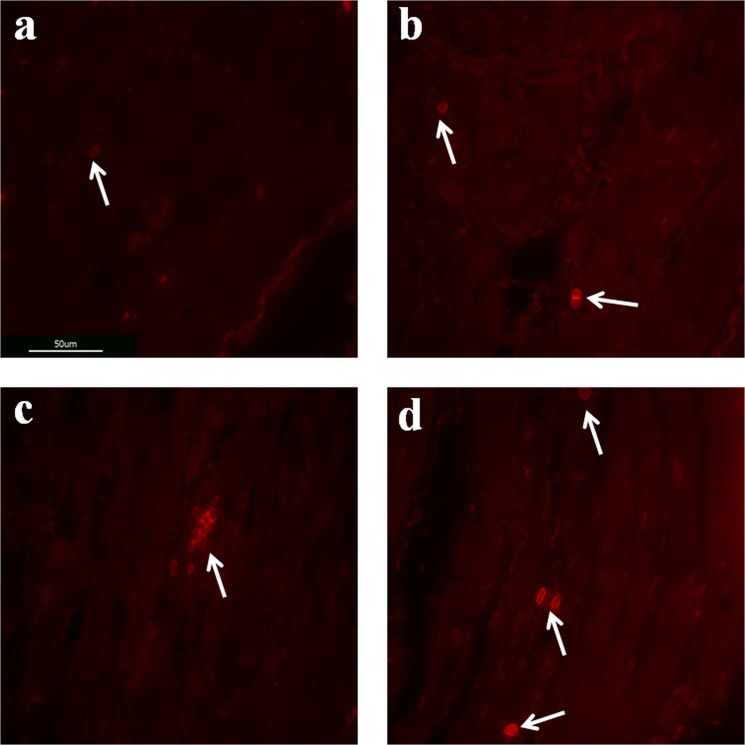
Immunofluorescent stainings of human left atrial appendage tissues for CD3-positive T cells. Sections of left atrial appendage tissues demonstrating the presence of CD3-positive T cells (1 cell, white arrow) in patients with sinus rhythm (**a**), paroxysmal (3 cells, white arrows) atrial fibrillation (**b**), peristent (7 cells, white arrow) atrial fibrillation (**c**) and permanent (4 cells, white arrows) atrial fibrillation (**d**). Whereas the number of CD3-positive T cells increased from sinus rhythm over paroxysmal atrial fibrillation to persistent atrial fibrillation, cell counts subsequently decreased from persistent to permanent atrial fibrillation.

The number of inflammatory cells identified as CD3-positive T cells in the left atrial appendage tissues in the AF groups was generally significantly higher than the number of inflammatory cells in the left atrial appendage tissues in the SR group (p < 0.001, Fig. 2). When samples from the SR group and the three individual subgroups of AF patients were compared, the number of inflammatory CD3-positive T cells was significantly higher in patients with paroxysmal AF than in the SR group (p < 0.001) and significantly higher in patients with persistent AF than in the subgroup of paroxysmal AF (p = 0.003). Interestingly, in patients with persistent AF, these cells were frequently arranged in small clusters (Fig. 3C). Subsequently, the number of inflammatory CD3-positive T cells decreased and was significantly lower in patients with permanent AF than in patients with persistent AF (p < 0.001). The amount of inflammatory CD3-positive T cells did not differ between the subgroups of paroxysmal and permanent AF (p = 0.502) (Figs. 2 and 3). In absolute numbers, the average count of CD3-positive T cells per 1 mm² was 0.27 ± 0.05 in the SR group, 1.55 ± 0.27 in patients with paroxysmal AF, 2.38 ± 0.39 in the subgroup of persistent AF and 1.28 ± 0.19 in patients with permanent AF, respectively. Regarding the localization, the endo- and subendomyocardium were generally more subjected to the infiltration of CD3-positive T cells than the midmyocardium. Inflammatory CD20-positive B cells could only be detected very occasionally in all AF subgroups and were not locatable in patients with SR. They were only rarely found in the subendomycardium as singular cells and were undetectable in the midmyocardium.

## Discussion

This study examined the association of inflammatory B (CD 20-positive) and T (CD3-positive) cell infiltration in left atrial appendage tissue of patients with SR and different stages of AF. Whereas inflammatory CD20-positive B cells could only be detected very occasionally in all AF subgroups, this study is the first to demonstrate significantly ascending inflammatory CD3-positive T cell counts over the course of SR to paroxysmal AF and persistent AF. Interestingly, a subsequent abatement of inflammatory CD3-positive T cells from persistent to permanent AF could be detected.

In how far inflammation is a cause of AF or solely a consequence is still a matter of debate. Expressions of both CD69 and HLA-DR on peripheral blood CD3-positive T lymphocytes as markers of T cell activation have been shown to be significantly higher in patients with AF than in individuals with SR^20^. Moreover, previous studies have demonstrated increased inflammatory markers in serum or plasma in AF patients. For instance, CRP, IL-2, IL-6, IL-8 and TNFα have been found as related with AF and its outcome^12^. However, results regarding the level or the increase of inflammatory markers and the natural history of AF are ambiguous. Likewise in our study, most of them seem not to increase in the context of transition from earlier to later stages of AF. More importantly, we generally did not observe any significant differences in CRP or IL-6 concentrations and leukocytes between patients with SR or AF. Some previous studies have indicated that serum concentrations of TNFα and highly sensitive (hs)-CRP might show a graduated increase from paroxysmal to persistent and permanent AF^21,22^. Additionally, serum concentrations of IL-6, a cytokine with pleiotropic effects on inflammation, were found to be increased in AF patients compared with healthy individuals^11^. In line with these prementioned findings, in our study there was a tendency towards higher levels of CRP and IL-6 in patients with persistent and permanent AF, respectively. Another reason for this might be the fact that CRP and IL-6 levels were also shown to be higher in blood samples drawn from patients during AF than those from patients during SR^23^. In this regard, in all of our patients with paroxysmal AF blood samples were collected during SR, whereas in the majority of patients with persistent AF the arrhythmia could be detected electrocardiographically at the time of study inclusion. However, to date there are generally contradictory results to what extent this evidence of inflammation reflects AF or merely a potential underlying disease. Considering these previous ambiguous findings in literature and the data in our study, the exact mechanism responsible for increased circuclating inflammatory markers remains indeterminante and it is unclear whether the aforementioned results conceivably represent active participation of local inflammatory processes during AF or a sort of systemic inflammation in consequence of concomitant diseases.

Apart from analysis of systemic inflammatory markers, there have been only few histological studies in the past that investigated the relation between AF and inflammation and provided direct local verification of a link between these entities. Yamashita *et al*. observed adhesion and migration of CD45-positive cells in left atrial appendages of patients with AF, which most of them were immunologically active CD68-positive macrophages, whereas CD3-positive T cells infiltrated to a lesser extend^18^. Another study also revealed an infiltration of inflammatory CD45-positive cells in the right and left atrial myocardium of AF patients^19^. Most recently, Smorodinova *et al*. reported on elevated numbers of CD3-positive T cells and CD68-KP1+ dendritic cells in bioptic samples of the left atrial myocardium of patients with AF compared to those with SR^17^.

In fact, our results are generally in line with the previous data and support the presence of inflammatory CD3-positive T cells in human left atrial tissues of patients with AF. However, the present study extends these findings in relation to two notable aspects. First, we examined the presence of inflammatory CD20-positive B cells using immunohistochemical analysis in whole left atrial appendage tissues and not only in bioptic samples of patients with AF and SR. In comparison to CD3-positive T cells, this cell type could be detected only very occasionally in all AF subgroups and was not locatable in patients with SR. Secondly and more importantly, this is the first study demonstrating that the infiltration of CD3-positive T cells seems to be affected by AF duration as statistically significant increasing cell counts could be detected between patients with SR, paroxysmal AF and persistent AF, respectively. Furthermore, the number of inflammatory CD3-positive T cells subsequently decreased and was significantly lower in patients with permanent AF than in patients with persistent AF. Hence, it is tempting to speculate that over the course and duration of the disease local inflammatory processes are particularly accentuated at earlier stages, whereas structural remodeling, namely atrial stretching leading to collagen deposition and subsequent interstitial fibrosis, are the predominant processes at later stages of the disease.

In accordance with a previous survey, in our cohort CD20-positive B cells were only occasionally detected in all AF subgroups. In the past, autoantibodies such as anti-β1-R, anti-M2-R, anti-sodium-potassium pump autoantibody and anti-heat shock protein (anti-HSP) autoantibody were found to be elevated in AF patients^24,25^. Regarding the latter, in a prospective study of 329 patients undergoing elective coronary artery bypass grafting surgery, the presence of increased anti-HSP65 in blood samples was independently associated with postoperative AF^26^. In another study, higher anti-HSP70 antibody levels were recorded in patients with persistent AF than in their counterparts with paroxysmal AF^27^. Moreover, also the strikingly elevated risk of AF in patients with certain autoimmune diseases indicates a pathogenic role of humoral immune responses^28^. Recently, anti-M2-R antibody levels in paroxysmal lone AF patients could strongly be correlated with the extent of LA fibrosis^29^. However, ongoing studies addressing the underlying mechanisms of a humoral immune response in AF are lacking. A possible explanation for the rare detection of CD20-positive B cells in our cohort and in a previous survey might be the assumption that circulating antibodies or their local deposition rather than the presence of CD20- positive B cells themselves represent the pathologic substrate in the development and perpetuation of AF.

Earlier studies have indicated that broad anti-inflammatory modulation with steroids or vitamin C substantially reduces both AF episodes and the risk of developing AF^13,15,16^. In order to address the enormous health care burden associated with AF it is of particular need to enhance the understanding of AF epidemiology and to develop novel medicative therapeutic strategies. In this respect and in connection with our findings, further treatment of the inflammatory pathway in terms of a targeted therapy with monoclonal anti-CD3 antibodies may appear as a suitable means at early stages of the disease. However, a recent randomized open-label multicenter phase I/II trial with a monoclonal anti-CD3 antibody in patients with severe steroid-refractory ulcerative colitis has demonstrated that apart from symptomatic response and symptomatic remission rates almost all participants experienced adverse events or serious adverse advents, which included abdominal abscess, cytomegalovirus infection, herpes zoster, esophageal candidiasis or even atrial fibrillation in some cases^30^. These findings underline the complex nature and interaction of inflammation in the pathophysiology of AF in which the involvement of inflammatory CD3-positive T cells appears to be one important component.

Taken together, our data emphasize the potential prominent role of the cellular component of the immune system in the development and perpetuation of AF. However, further studies are needed to determine the temporal succession and coherence among inflammation and AF duration especially with regard to the development of potential new treatment strategies.

### Study limitations

A possible restriction of this study is that our analysis focused on the differences between patients with SR and paroxysmal, persistent or permanent AF undergoing surgery for heart valve repair or replacement in the majority of cases. Thus, it might be suggested that the valvular disease itself could be the origin for local inflammation within the atrial myocardium. However, the different extent of immune cell infiltration in our study can hardly be explained only by this factor. Second, due to the exclusive focus on left atrial appendage tissue we cannot provide data about the presence of inflammatory cells in other parts of the atrium. However, in a previous study a good correlation between the number of inflammatory cells in the right and left atrial myocardium could be demonstrated^19^. Third, our analyses only focused on CD3-positive T cells and CD-20-positive B cells. Therefore, no specifications can be made about the presence and potential influence of other subgroups of the cellular immune system. Lastly, the relatively small size of our patient group might affect our quantitative results and statistical analyses.

## Data Availability

Material, data and associated protocols are available from the corresponding author upon request.
